# 2-Phenylethyl Isothiocyanate Exerts Antifungal Activity against *Alternaria alternata* by Affecting Membrane Integrity and Mycotoxin Production

**DOI:** 10.3390/toxins12020124

**Published:** 2020-02-15

**Authors:** Miao Zhang, Yongcai Li, Yang Bi, Tiaolan Wang, Yupeng Dong, Qian Yang, Tingting Zhang

**Affiliations:** College of Food Science and Engineering, Gansu Agricultural University, Lanzhou 730070, China; Zhangmiao321@hotmail.com (M.Z.);

**Keywords:** 2-phenylethyl isothiocyanate, *Alternaria alternata*, pear fruit, black spot, membrane integrity, mycotoxin

## Abstract

Black spot caused by *Alternaria alternata* is one of the important diseases of pear fruit during storage. Isothiocyanates are known as being strong antifungal compounds in vitro against different fungi. The aim of this study was to assess the antifungal effects of the volatile compound 2-phenylethyl isothiocyanate (2-PEITC) against *A. alternata* in vitro and in pear fruit, and to explore the underlying inhibitory mechanisms. The in vitro results showed that 2-PEITC significantly inhibited spore germination and mycelial growth of *A. alternata—*the inhibitory effects showed a dose-dependent pattern and the minimum inhibitory concentration (MIC) was 1.22 mM. The development of black spot rot on the pear fruit inoculated with *A. alternata* was also significantly decreased by 2-PEITC fumigation. At 1.22 mM concentration, the lesion diameter was only 39% of that in the control fruit at 7 days after inoculation. Further results of the leakage of electrolyte, increase of intracellular OD_260_, and propidium iodide (PI) staining proved that 2-PEITC broke cell membrane permeability of *A. alternata*. Moreover, 2-PEITC treatment significantly decreased alternariol (AOH), alternariolmonomethyl ether (AME), altenuene (ALT), and tentoxin (TEN) contents of *A. alternata*. Taken together, these data suggest that the mechanisms underlying the antifungal effect of 2-PEITC against *A. alternata* might be via reduction in toxin content and breakdown of cell membrane integrity.

## 1. Introduction

*Alternaria alternata* is a filamentous fungus [1] that causes different diseases during the postharvest shelf-life of horticultural products including black rot of cherry tomato [2], moldy core and core rot of apple [3], *Alternaria* brown spot of citrus [4], black rot of *Hylocereus undatus* [5], and *Alternaria* rot of netted melon [6]. *A. alternata,* the causal agent of black spot in pear, initially infects the fruit via styles or peel during the growing season and remains latent until fruit maturity [7], which results in severe economic and industrial losses [8]. Most *Alternaria* species produce mycotoxins during growth that pose potential threat to animal and human health [9]. These mycotoxins belong to three classes including dibenzo-α-pyrone derivatives (alternariol, AOH; alternariol monomethyl ether, AME; and alteuene, ALT), tetramic acid derivatives (tenuazonic acid, TeA), and perylene derivatives (altertoxin I, ATX-I; and the related compounds altertoxins II and III) [10,11]. Therefore, inhibiting mycotoxin synthesis or reducing mycotoxin levels are main fields of research interest in controlling postharvest black spot disease in fruit and vegetables. Recently, chemical fungicides (orthophenylphenate and imazalil) were found to be effective in controlling *A. alternata* [12]; however, the continued use of these fungicides has led to the emergence of resistant strains and has generated environmental pollution and food security issues [13,14]. Therefore, it is necessary to find safe and effective natural alternatives to chemical fungicides to control postharvest diseases of pear fruit.

Vegetables of the genus *Brassica* such as cauliflower, broccoli, cabbage, canola, kale, turnip, radish, and various mustards produce a good deal of potentially antimicrobial compounds called glucosinolates (GSLs) [15,16]. Isothiocyanates are one of the products of GSL hydrolysis by the enzyme myrosinase (thioglucosidase) [17]. They mainly include phenethyl isothiocyanate (PEITC), allyl isothiocyanate (AITC), benzyl isothiocyanate (BITC), erucin (ERU), phenylhexyl isothiocyanate (PHI), and sulforaphane (SFN) [18]. Studies have shown that these isothiocyanates reduce the risk of some types of cancer [19,20] and cardiovascular diseases [21]. Furthermore, isothiocyanates have demonstrated potential inhibitory activity against fungi [22], nematodes [23,24], bacteria [25,26], insects [27], and weeds [28]. Isothiocyanates have been reported to possess fungistatic and fungitoxic activities against a variety of plant pathogens [29]. Studies have demonstrated fungicidal activity for various compounds as follows: AITC against *Monilinia laxa* of nectarine and peach [30], *Botrytis cinerea* of strawberry [31], and *Aspergillus parasiticus* of pizza crust [32]; BITC against *Aspergillus ochraceus* of peanuts, green coffee, grapes, and soybeans [33], *Phymatotrichopsis omnivora* of cotton [34], and *A. alternata* of tomato [35]; and 2-propenyl-isothiocyanate against *Penicillium expansum* of pears [36]. However, the inhibitory effect depends on the type and concentration of isothiocyanate, the type of fungus, application method, and time of exposure. Among them, 2-phenylethyl isothiocyanate (2-PEITC), a major isothiocyanate, is the most common glucosinolate in *Brassica* species. Studies have shown that the antimicrobial activity of 2-PEITC against soil microorganisms [37], plant pathogenic bacteria [38], and clinical isolates. However, the role of 2-PEITC on fungal pathogens in postharvest fruit and its mode of action are largely unknown. The aim of the present study was to evaluate the inhibitory effects of 2-PEITC on *A. alternata* both in vitro and in pear fruit, as well as to further ascertain the antifungal mechanisms of 2-PEITC by analyzing membrane integrity and toxin production.

## 2. Results

### 2.1. Effects of 2-PEITC on Spore Germination and Mycelial Growth of A. alternata

**Figure 1:**
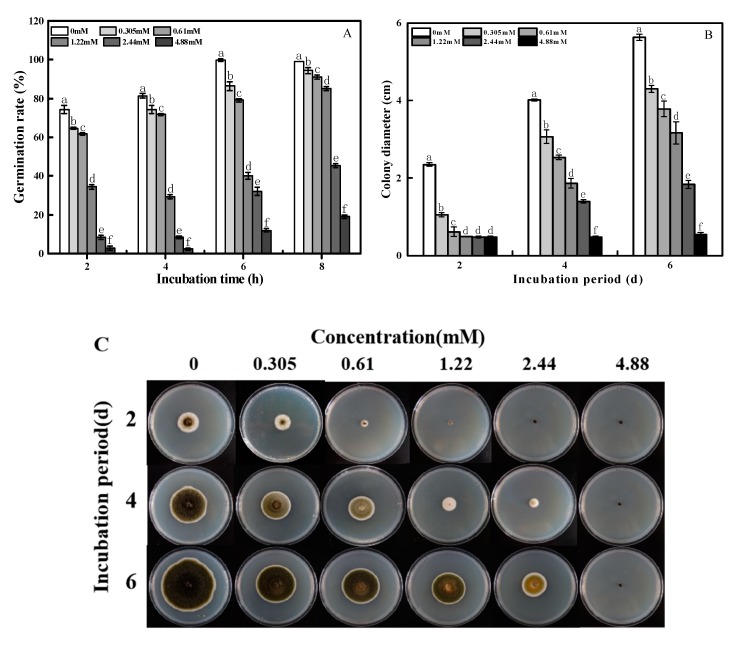
Effects of 2-phenylethyl isothiocyanate on spore germination rates (**A**), and the mycelial diameter (**B**) and colony morphology (**C**) of *A. alternata.* Colony diameter was examined every 2 days after incubation at 28 °C for a total of 6 days. Treatments followed by different letters are significantly different according to Duncan’s multiple range test (*p* < 0.05).

The spore germination rate of *A. alternata* was significantly inhibited after 2-PEITC fumigation, and a positive correlation (*R*^2^ = 0.8979, *p* < 0.05) was observed between the concentration of 2-PEITC and the inhibitory effect (Figure 1A). 2-PEITC at 0.305 mM inhibited spore germination of *A. alternata* after incubation for 2 h. At 4.88 mM, the rate of spore germination was inhibited by 81% after 8 h of incubation. 

2-PEITC effectively inhibited the mycelial growth of *A. alternata*, and the inhibitory effect showed a dose-dependent manner (Figure 1B,C); 2-PEITC at 1.22 mM or higher concentrations entirely inhibited the mycelial growth of *A. alternata* 2 days after treatment. Thus, the minimum inhibitory concentration (MIC) of 2-PEITC was 1.22 mM.

### 2.2. Inhibition of on the Lesion Development of Black Spot in Pear Fruit

**Figure 2:**
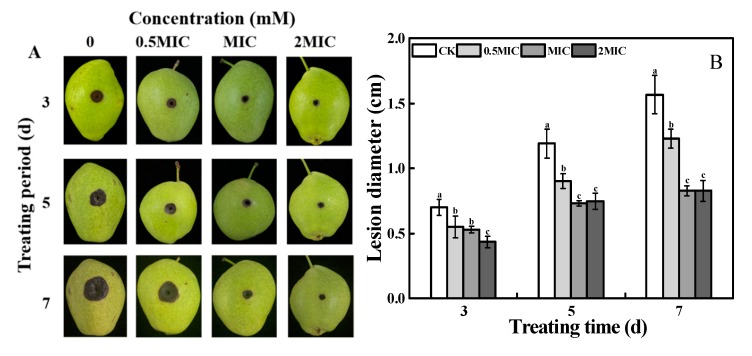
Disease severity of spot disease caused by *Alternaria alternata* in pear fruits stored at 20 ± 2 °C and efficacy of 2-phenylethyl isothiocyanate at various concentrations. (**A**) Pear fruits at 3, 5, and 7 days of storage. (**B**) Statistical analysis of lesion diameter shown as histograms. Treatments followed by different letters are significantly different according to Duncan’s multiple range test (*p* < 0.05).

As shown in Figure 2, 2-PEITC treatment significantly controlled black spot caused by *A. alternata* in pear fruit. The disease severity, indicated by the lesion diameter of black spot, was effectively reduced by 2-PEITC in a dose-dependent manner. The lesion diameter was reduced after 2-PEITC application at a concentration of 0.61 mM. At 1.22 mM (MIC), the lesion diameter was only 39% of that in the control fruit at 7 days after inoculation (Figure 2A,B). However, control effects between MIC and higher concentrations (2 × MIC) of 2-PEITC fumigation showed no significant difference.

### 2.3. 2-PEITC Damaged Plasma Membrane Integrity of A. alternata

**Figure 3:**
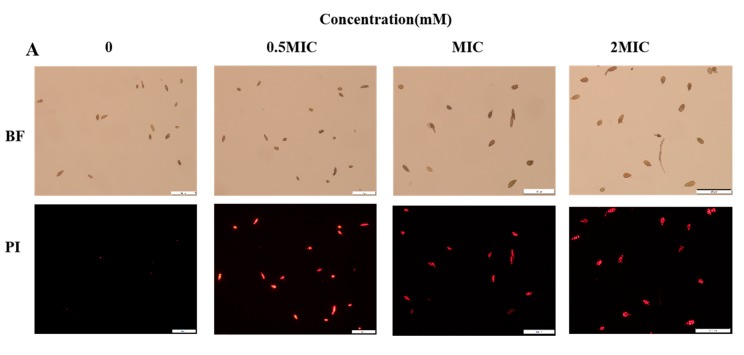

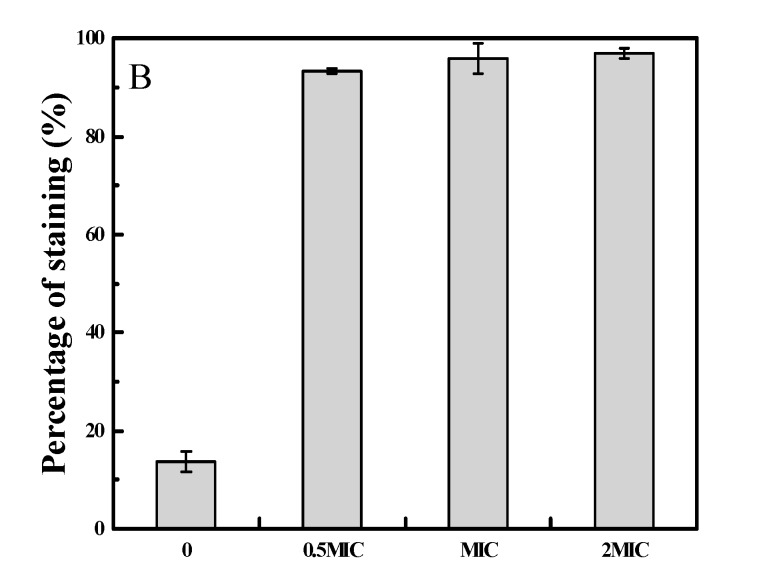
Plasma membrane integrity of *A. alternata* treated with 2-phenylethyl isothiocyanate at various concentrations. (**A**) Propidium iodide (PI) staining assay. Scale bars: 100 μm. (**B**) percentage of PI staining. Red fluorescence indicates spores with disrupted plasma membranes. Bars represent 100 μm.

To further explore the potential antifungal mechanisms of 2-PEITC, propidium iodide (PI), which has the ability to permeate broken cell membrane and emit red fluorescence, was used to test plasma membrane integrity in *A. alternata* cells. As shown in Figure 3A, mycelia of the control were not stained with PI, and low red fluorescence was observed; however, after 2-PEITC treatment, the fluorescence obviously increased, and 97% of MIC 2-PEITC-treated cells were stained with PI (Figure 3B).

### 2.4. Cellular Leakage of A. alternata after 2-PEITC Treatment

**Figure 4:**
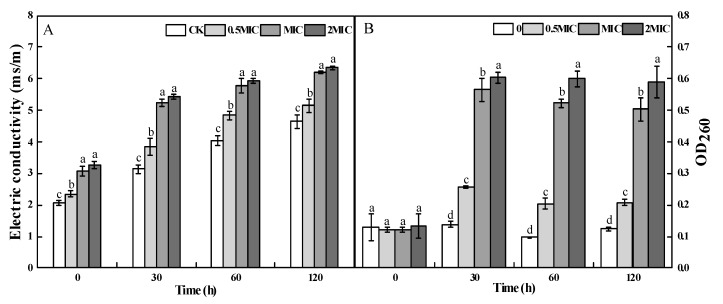
Effect of 2-phenylethyl isothiocyanate on electrolyte leakage (**A**) and nucleic acid content (**B**) of *A. alternata*. Treatments followed by different letters are significantly different according to Duncan’s multiple range tests (*p* < 0.05).

Electrolyte leakage and nucleic acid content were used to determine the permeability of plasma membrane. 2-PEITC treatment clearly increased the electric conductivity of *A. alternata.* After 120 min of incubation, electric conductivity of 0.5 × MIC, MIC, and 2 × MIC 2-PEITC-treated *A. alternata* cells was increased by 10%, 33%, and 37% compared with the control, respectively (Figure 4A). Similarly, leakage occurred after 30 min of 2-PEITC treatment and nucleic acid content increased after 2-PEITC treatment. After 30 min treated by 2-PEITC, the OD_260_ of *A. alternata* was the highest, and the OD_260_ in the 2-PETIC-treated *A. alternata* with concentrations of 0.5 × MIC, MIC, and 2 × MIC were 1, 3.08, and 3.37 times higher than the control, respectively (Figure 4B).

### 2.5. Production of Mycotoxins by A. alternata upon 2-PEITC Treatment

**Figure 5:**
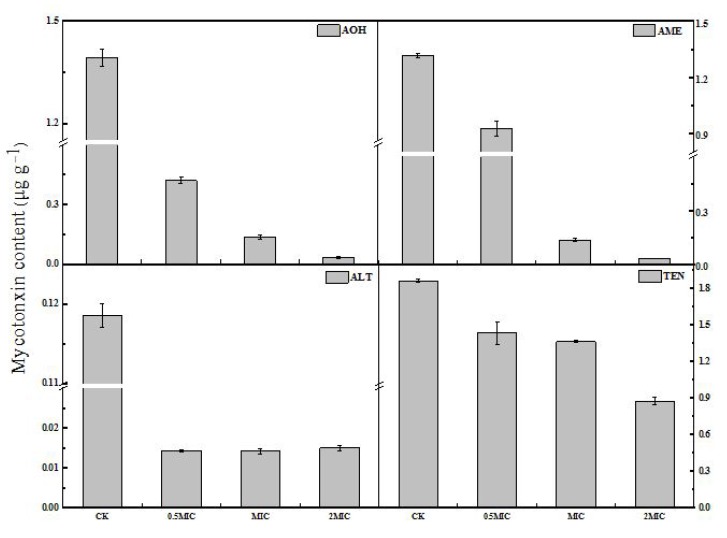
Alternariolmonomethyl ether (AME), alternariol (AOH), altenuene (ALT), and tentoxin (TEN) contents of *A. alternata* treated with 2-phenylethyl isothiocyanate. The amounts of the metabolites were determined by HPLC using triplicate samples and are represented as means ± standard deviations.

Four mycotoxins including tentoxin (TEN), AOH, AME, and ALT were extracted and detected by high performance liquid chromatography-tandem mass spectrometry (HPLC-TOF-ESI-MS) in the mycelium of *A. alternata*. As shown in Figure 5, the contents of TEN, AOH and AME in *A. alternata* hypha significantly decreased with the increase of 2-PEITC concentration. The concentrations of TEN, AOH, AME, and ALT in MIC of 2-PEITC-treated groups were only 27%, 90%, 90%, and 88% of the corresponding control groups, respectively.

## 3. Discussion

All kinds of plant-derived antifungal compounds such as geraniol, citral, octanal, cinnamaldehyde, and different essential oils, which have potential in targeting fungal pathogens in fresh fruit and vegetables, have been extensively studied [39,40,41,42]. Due to the emergence of these natural compounds that bear strong antifungal properties, the use of synthetic fungicides can be reduced. Isothiocyanates, a group of defense-related compounds synthesized by plants of the genus *Brassica,* are promising alternatives to synthetic chemical fungicides. Evidence has shown that different isothiocyanates possess strong antifungal activity against *B. cinerea*, *Rhizopus stolonifer*, *M. laxa*, *Mucor piriformis,* and *Penicillium expansum* [29]. Mari et al. [43] reported that 3-methylsulfinil-3-butenyl-isothiocyanate at a low concentration of 0.02 g L^−1^ inhibited *M. laxa*, whereas 0.93 g L^−1^ of the same compound was needed to inhibit *P. expansum*; p-hydroxybenzyl-isothiocyanate inhibited *M. laxa* and *P. expansum* at 0.09 g L^−1^ concentration; however, 1.8 g L^−1^ was needed to inhibit *R. stolonifer*. In addition, 2-PEITC inhibited mycelial growth of *Sclerotinia sclerotiorum* in a dose-dependent manner with 100% inhibition at a concentration of 0.67 mM [15]. Using the same biofumigation method and exposure time, we observed that 2-PEITC at 1.22 mM or higher concentrations entirely inhibited the mycelial growth of *A. alternata* 2 days after treatment (Figure 1), which indicates that the inhibitory effect depends on the type and concentration of the isothiocyanate. Spore germination of *A. alternata* was also significantly inhibited by 2-PEITC treatment; however, 100% inhibition was not recorded even at the highest concentration tested (Figure 1A), which indicates that the spore of *A. alternata* is more resistant to 2-PEITC than the mycelium. A similar result reported by Harvey et al. [44] showed 50% and 90% inhibition of spore germination in *Sclerotium rolfsii* by 2-propenyl isothiocyanate at 249 and 528.8 mM, respectively. Moreover, AITC exposure in strawberries infected by *B. cinerea* reduced fruit decay, which indicates that isothiocyanates improve plant defense against microbial pathogens [31]. Results presented in this study showed that 2-PEITC at MIC efficiently decreased black spot disease severity in pear fruit. However, 2-PEITC at 3.06 mM significantly reduced rot of netted melon inoculated with *A. alternata* [6], which may have been due to the difference in susceptibility of different fruit to 2-PEITC.

Cell membrane is important for the growth and reproduction of microorganisms [45]. The complete cell membrane system is the basis of biophysiological activities. Some studies have suggested that antifungal activity of volatile compounds could be closely correlated with cell membrane integrity of microorganisms [46]. Ji et al. [47] detected loss of membrane integrity in *B. cinerea* after methyl thujate treatment (60 g L^−1^). Membrane permeability of *Penicillium cyclopium* increased with increasing concentrations of E-2-hexena, evidenced by cell constituent release and leakage of potassium ions [41]. The current study found the red fluorescence levels in *A. alternata* cells and fluorescence value in the fungal suspensions visibly increased with 2-PEITC treatment (Figure 3A); the results deduced that 2-PEITC disorganized and disrupted membrane order and integrity. In addition to destroying the plasma membrane, natural compounds could alter transport process in fungi and induce the leakage of intracellular ions [39,48]. In the present study, extracellular conductivity increased rapidly after treatment with MIC of 2-PEITC. The maximum extracellular conductivity was examined in *A. alternata* cell suspension treated with 2-PEITC after 120 min of incubation (Figure 4A). Meanwhile, a significant increase in nucleic acid leakage was observed after 2-PEITC treatment (Figure 4B). These results further prove that the antifungal activity of isothiocyanates might be due to the disruption of cell membrane integrity in *A. alternata.*

Mycotoxins are common virulence factors in the host infected by plant pathogens [49]. *Alternaria* toxins have been shown to exhibit serious effects, and therefore pose a threat to animal and human health. *A. alternata* is involved in the production of mycotoxins, including AOH, AME, ALT, TEN, and altertoxins (ATX) in fruit and vegetables [50,51]. Some studies have reported that volatile compounds affect the synthesis of toxins. Quiles et al. [32] reported that AITC inhibits the *Aspergillus parasiticus* growth and aflatoxin (AF) production. However, some studies found that volatile compounds only reduced the amount of mycotoxins. The effects of BITC on *Aspergillus ochraceus* morphology were related to a decrease in ochratoxin (OT) production, both ochratoxin A (OTA) and ochratoxin B (OTB) [33]. In the present study, we found that the contents of TEN, AOH, AME, and ALT with the 2-PEITC-treated group significantly decreased compared to the control. This finding agrees with Xu et al. [52], who reported that AOH and AME in *A. alternata* were degraded after cinnamaldehyde treatment (0.200 mL L^−1^) and incubation for 120 min. However, the underlying mechanism 2-PEITC employed to modulate toxin synthesis in *A. alternata* needs to be further explored.

Although results from the present study and several previous studies showed that isothiocyanates including 2-PEITC derived from plants are biofumigant fungicides against postharvest pathogens such as *S. sclerotiorum* [15], *B. cinerea* [31], and *P. expansumon* [36] through inhibiting vegetative growth, damaging cell membrane, and impairing mycotoxin synthesis, among other factors, most studies only provided experimental evidence. However, isothiocyanates are very low in plant material, and thus a large amount of plant material is needed to isolate sufficient quantities to meet warehouse storage fumigation. In addition, the application of isothiocyanates is limited due to their strong odors, which significantly affect the taste of fruit and vegetables. Thus, some integrated techniques including preparing isothiocyanate mixture [53] and isothiocyanate microcapsules [11,54,55] have been recently explored for their practical application. Meanwhile, the application effect of isothiocyanates depends on the postharvest diseases to be controlled, the application method, the fumigation time, and the type and concentration of isothiocyanates. Therefore, feasible and cost-effective techniques of 2-PEITC application for postharvest disease control need to be further explored.

## 4. Conclusions

In conclusion, this work elucidated 2-PEITC as an effective antifungal compound to control postharvest black spot disease caused by *A. alternata* in pear fruit, and the MIC was 1.22 mmol/L after in vitro experiment. Moreover, 2-PEITC disrupted the permeability and integrity of the fungal membrane that led to leakage of cytoplasmic contents and finally cell death. In addition, 2-PEITC treatment significantly decreased the content of mycotoxins, including TEN, AOH, AME, and ALT, in *A. alternata.* These results suggest that 2-PEITC could be an alternative fungicide for the control of black spot in pear fruit caused by *A. alternata.*

## 5. Materials and Methods

### 5.1. Fruit and Pathogens

“Zaosu” pear (*Pyrus bretschneideri* Rehd) were commercially picked from the Tiaoshan Farm in Jingtai County, Gansu Province, China. Pears of uniform size, with no mechanical damage, no pests, and diseases were selected, and the fruit were individually packaged in a plastic mesh bag, transported the laboratory, and stored at 4 °C. A total of 28 pears were required for this experiment.

The preparation of spore suspension was in reference to Tahir et al. [56]. A total of 10 mL sterile distilled water containing 0.01% (v/v) Tween-80 was poured into the 7 day *A. alternata* culture dish under sterile conditions. The spores were scraped with a sterilized applicator and filtered through four layers of sterile cheesecloth and vortexed for 20 s, and the suspension was then adjusted to the required number of spores with a hemocytometer.

### 5.2. Mycotoxin Standards and Chemicals

AOH, AME, ALT, and TEN were purchased from Pribolab (Pte. Ltd. Singapore) and stored at −20 °C for HPLC analysis. 2-PEITC was purchased from Shanghai Macklin Biochemical Co., Ltd. (Shanghai, China).

### 5.3. In Vitro Inhibitory Effects of 2-PEITC on Growth of A. alternata

Mycelial growth of *A. alternata* was measured according to a previously reported method [57]. In short, 2 μL spore suspension (1 × 10^5^ CFU mL^−1^) was dripped onto the center of each Petri dish mounted with potato dextrose agar (PDA) medium. The amount of 2-PEITC was calculated according to the space of the Petri dish (0.0635 mm^3^), and a sterilized filter paper disc (20 mm diameter) was attached on the center of the lid inner surface with different concentrations of 2-PEITC (0.305, 0.61, 1.22, 2.44, and 4.88 mM; the unit was defined as 2-PEITC volume per the Petri dish space) added onto the paper, and then the plate was quickly covered. Filter paper without 2-PEITC was used as the control. After the plates were incubated at 28 ± 1°C for 2, 4, and 6 days, the diameter (cm) of the colony area was determined. Each treatment comprised three replicates. The lowest concentration in which no visual hyphal growth was observed after 2 days of incubation was the minimum inhibitory concentration (MIC).

To evaluate the effects of 2-PEITC fumigation on spore germination of *A. alternata*, the spore suspension (1 × 10^5^ CFU mL^−1^) was dripped onto the slide and placed in a large Petri dish with wet filter paper (humidity = 95%). The amount of 2-PEITC was calculated according to the space of the Petri dish (0.1905 mm^3^), and a sterilized filter paper disc (20 mm diameter) was attached on the center of the lid inner surface with different concentrations of 2-PEITC (0.305, 0.61, 1.22, 2.44, and 4.88 mM; the unit was defined as 2-PEITC volume per the Petri dish space) added onto the paper, and then the plate was quickly covered. Treatment without adding 2-PEITC was the control. Spore germination rate was observed and calculated after 2, 4, 6, and 8 h of incubation. Each treatment comprised three replicates.

### 5.4. Effect of 2-PEITC on A. alternata in Pear Fruit

The in vivo assay was carried out according to a previously described method with minor modifications [58]. Zaosu pear fruit were dipped into 2% sodium hypochlorite for 30 s, washed with distilled water and air-dried at room temperature, wounded on the epidermis in the equatorial region with a sterile punch (2 mm deep, 5 mm wide), and each wound site was inoculated with 20 μL of spore suspension at 1 × 10^6^ CFU mL^−1^. After 2 h incubation, the inoculated fruit were fumigated in plastic boxes (lid was drilled with air holes). The amount of 2-PEITC was calculated according to the space of the plastic boxes (200 × 130 × 50 mm), a sterilized filter paper disc (30 mm diameter) was attached on the center of the lid inner surface with different amount of 2-PEITC (0.5 × MIC, MIC, 2 × MIC) added onto the paper, and then the plate was quickly covered. Treatment without adding 2-PEITC was the control. Each treatment contained nine fruit. Fruit were fumigated in plastic boxes with humidity (55%) at room temperature (23 ± 1 °C) for 1 day and later taken out and stored in a paper carton (humidity = 55%) at room temperature. Disease severity was determined 3, 5, and 7 days after the treatment by measuring the lesion diameter.

### 5.5. Determination of Plasma Membrane Integrity

Membrane integrity of *A. alternata* cells exposed to 0.5 × MIC, MIC, and 2 × MIC 2-PEITC was a slightly modified version of a previously described method [59]. Initially, different 2-PEITC concentrations (0.5 × MIC, MIC, 2 × MIC) were added to the fungal suspension (1 × 10^6^ CFU mL^−1^), incubated in a moist chamber at 28 ± 1 °C for 30 min, centrifuged at 8000 rpm for 5 min, washed three times with phosphate-buffered saline (PBS, 0.05 M, pH 7.2), and stained with propidium iodide (PI) for 5 min at 30 °C. After centrifugation, fungal suspension was washed three times with PBS solution (0.05 M, pH 7.2) to remove the residual dye, and was observed under a fluorescence microscope (OLYMPUS CORPORATION, Olympus U-LH100HG, Tokyo, Japan) at an excitation wavelength of 620 nm.

### 5.6. Detection of Cellular Electrolyte Leakage and Nucleic Acid Content

Electrolyte leakage of *A. alternata* cells was measured according to a previously described method [60] with minor modifications. 2-PEITC (0.5 × MIC, MIC, 2 × MIC) was added to the fungal suspension (1 × 10^6^ CFU mL^−1^) and incubated for 0, 30, 60, and 120 min. Subsequently, the electric conductivity was evaluated in a conductivity meter (DDS-307, Hanghai Yueping Scientific Instrument Co., Ltd. Shanghai, China).

Nucleic acid content of the supernatant was measured following the method of Paul et al. [61] with some modifications. Mycelia of *A. alternata* culture grown for 4 days in 0.5 g PDA was collected and resuspended in 15 mL PBS (pH 7.0). The suspension was treated with 2-PEITC at different concentrations (0.5 × MIC, MIC, 2 × MIC) and incubated at 28 ± 1 °C for 0, 30, 60, and 120 min. Subsequently, the suspension was centrifuged at 12,000 rpm for 2 min and the supernatant was collected. Absorbance of the supernatant (1 mL) was evaluated at 260 nm with UV-2450 UV/VIS Spectrophotometer (Shimadzu). Each treatment comprised three replicates. The control group was calibrated with PBS (pH = 7.0).

### 5.7. Mycotoxin Extraction and HPLC-MS Analysis

For mycotoxin extraction, the fungi were cultured in PDA at 28 °C for 4 days and fumigated with 2-PEITC (0.5 × MIC, MIC, 2 × MIC), followed by accurately weighed 0.5 g of *A. alternata* mycelium liquid nitrogen grinding, subsequently the hyphae were transferred to 10mL sterile centrifuge tubes, Subsequently, 2.5 mL of acetonitrile and water extract (4:1 v/v) containing 0.3% formic acid was added. The solution was vortexed, and was then mixed with 2.5 r s^−1^ extraction at room temperature for 30 min. Then, 0.25 g anhydrous MgSO_4_ and 0.04 g NaCl were added, and the mixture was shaken vigorously for 1 min, followed by centrifugation at 180 r s^−1^ for 10 min at 4 °C. Finally, the supernatant was taken through the 0.22 micron organic filter, quasi-determined to 1.2 mL, and the sample was prepared for HPLC analysis [62].

Separation and qualitative analysis of TEN, AOH, AME, and ALT were performed using a mass spectrometer (Agilent 1290, Anjielun, Shenzhen, China) equipped with the electrospray ionization (ESI) source. HPLC conditions were as a follows: column, C18 (250 × 4.6 mm, 5 μm); column temperature, 35 °C; injection volume, 5 μL; mobile phase A was deionized water, mobile phase B was methanol; gradient elution conditions were A after 70% retention for 1 min, after falling to 50% within 2 min, continued to drop to 10% within 1 min, kept for 2 min, rose to 90% within 0.1 min, kept for 2 min; tassel 0.005 mL s^−1^; total running time, 7.1 min.

Following the mass spectrometry conditions: ion source mode, positive ion mode (ESI^+^); mass spectrometry scanning mode, multiple reaction monitoring (MRM); sheath gas temperature, 350 °C; nebulizer pressure, 35 psi; sheath gas flow, 0.183 L s^−1^; capillary voltage, 4000 V; other parameters were adjusted to optimum by the instrument. The mass spectrometric parameters such as monitoring ion, cone voltage, and collision voltage of four *Alternaria* species are shown in Table 1.

### 5.8. Statistical Analysis

All data were expressed as mean ± SD by measuring three independent parallel replicates. The significance of the mean difference between the treatments at the 5% level was calculated by Duncan’s test using SPSS (version 13.0, SPSS Inc., Chicago, IL, USA).

## Figures and Tables

**Table 1:** Optimized multiple reaction monitoring (MRM) parameters for AOH, AME, ALT, and TEN mycotoxins.

Target Compounds	Ionization Mode	Parent Ion	Qualitative Ion	Keep Time	Quantitative Ion	Fragmentation Voltage	Collision Voltage
Altemariol (AOH)	ESI^+^	257.0	213.0	2.37	147.2	40	32
Altermariol monomethylether (AME)	ESI^+^	271.0	256.0	2.85	228.0 212.9	32	20
Allenuene (ALT)	ESI^+^	293.1	257.2	3.33	239.1	85	15
Tentoxin (TEN)	ESI^+^	415.2	312.3	3.66	189.0	110	30
